# Segment-Specific Adhesion as a Driver of Convergent Extension

**DOI:** 10.1371/journal.pcbi.1004092

**Published:** 2015-02-23

**Authors:** Renske M. A. Vroomans, Paulien Hogeweg, Kirsten H. W. J. ten Tusscher

**Affiliations:** 1 Theoretical Biology and Bioinformatics, Utrecht University, Utrecht, The Netherlands; Princeton University, UNITED STATES

## Abstract

Convergent extension, the simultaneous extension and narrowing of tissues, is a crucial event in the formation of the main body axis during embryonic development. It involves processes on multiple scales: the sub-cellular, cellular and tissue level, which interact via explicit or intrinsic feedback mechanisms. Computational modelling studies play an important role in unravelling the multiscale feedbacks underlying convergent extension. Convergent extension usually operates in tissue which has been patterned or is currently being patterned into distinct domains of gene expression. How such tissue patterns are maintained during the large scale tissue movements of convergent extension has thus far not been investigated. Intriguingly, experimental data indicate that in certain cases these tissue patterns may drive convergent extension rather than requiring safeguarding against convergent extension. Here we use a 2D Cellular Potts Model (CPM) of a tissue prepatterned into segments, to show that convergent extension tends to disrupt this pre-existing segmental pattern. However, when cells preferentially adhere to cells of the same segment type, segment integrity is maintained without any reduction in tissue extension. Strikingly, we demonstrate that this segment-specific adhesion is by itself sufficient to drive convergent extension. Convergent extension is enhanced when we endow our in silico cells with persistence of motion, which in vivo would naturally follow from cytoskeletal dynamics. Finally, we extend our model to confirm the generality of our results. We demonstrate a similar effect of differential adhesion on convergent extension in tissues that can only extend in a single direction (as often occurs due to the inertia of the head region of the embryo), and in tissues prepatterned into a sequence of domains resulting in two opposing adhesive gradients, rather than alternating segments.

## Author Summary

The process of convergent extension is a major contributor to the formation of the anterior-posterior body axis in the early embryo. Convergent extension refers to the directed movement of cells that leads to the extension of tissue in one direction and narrowing of the tissue in the perpendicular direction. Often, convergent extension occurs in tissue which already contains distinct domains of gene expression such as segments, and it is unclear how these patterns are maintained despite extensive cell movement. Interestingly, experimental evidence suggests that these tissue patterns may drive rather than be compromised by convergent extension. However, existing computational models aimed at unravelling the mechanisms of convergent extension have thus far only studied the process in homogeneous tissues. With our model, we demonstrate that in a segmented tissue, preferential adhesion of cells to other cells within the same segment type is required to maintain the tissue pattern during convergent extension. Furthermore, such segment-specific adhesion is by itself capable of driving convergent extension.

## Introduction

Convergent extension refers to the simultaneous narrowing and extension of tissues due to extensive cell rearrangements, and is a key morphogenetic event during formation of the bilaterian body plan. In bilaterian animals, convergent extension first occurs when the main body axis forms and extends, pushing the head and tail further away from each other. Although this axis extension is universal in bilaterians, the cell and tissue behaviour causing it differs widely between species (for reviews see [1–4]). In *Xenopus* for example, dorsal mesodermal cells polarize and change their adhesive properties (reviewed by [5]); cells then crawl between each other in a zipper-like process called intercalation [1, 2]. In contrast, convergent extension of zebrafish mesoderm consists of two processes: directed migration to the dorsal axis and intercalation [2–3, 6]. Finally, *Drosophila* germband extension occurs in a tightly connected epithelium, where cells intercalate by contracting those parts of the membrane that have a dorsal-ventral orientation [7–9].

Convergent extension is an inherently multiscale process, in which subcellular contractility and adhesion, cell level polarity and migration, and tissue level deformations are involved. Models incorporating this multiscale nature are of key importance to study the feedback interactions that give rise to tissue extension. Thus far, models are largely conceptual in nature, testing whether an experimentally observed (sub)cellular process or hypothetical mechanism can indeed drive convergent extension [10–13].

Among the identified mechanisms capable of driving convergent extension confirmed by these models are lamellipodia formation [13, 14], directed mitosis [13], oriented membrane contraction [8, 11], cell extension or protrusions [10–12] and anisotropic differential adhesion [15]. These different mechanisms also differ in the origin of the directional signal, the cue which informs cells into which direction to move. In the models including either directed mitosis, lamellipodia or oriented membrane contractions [11, 13, 14], this direction is explicitly imposed in the model by telling cells in which direction to extend. In contrast, in the models with anisotropic differential adhesion and cell elongation [12, 15], there is no global information: cells have internal polarity and through cell-cell interactions the cells align. Other models are somewhere in between; Rauzi *et al*. [8] use experimental data on the polar distribution of actomyosin, resulting in a coordinated contraction of only dorso-ventrally oriented membranes. The model by Weliky *et al*. [10] does not impose the direction in which cells extend, but includes two boundaries enclosing the tissue which inhibit cell extensions, thus providing an overall bias.

Regardless of how cell/tissue polarity is incorporated in these models, convergent extension has so far always been studied in homogeneous tissues consisting of cells with identical fates. However, axis extension usually does not occur in homogeneous tissues, but rather in tissues that have been or progressively become patterned into regions of different cell fate. In *Tribolium* for instance, segments are formed by an oscillating gene clock, shortly after which the newly segmented part of the tissue starts to narrow and extend [16–19]. Therefore, an interesting question is how patterns are maintained under convergent extension, which leads to extensive cell rearrangements and therefore potentially mixes up cells of different fate. Considering this, it is striking that in *Xenopus*, the antero-posterior patterning of the mesoderm is crucial for convergent extension [20]; also in *Drosophila*, a segmented body pattern is essential for germband extension [21, 22]. This leads to the intriguing suggestion that rather than segments becoming lost due to convergent extension, these segments may actively drive convergent extension.

Since the interplay between tissue patterns and convergent extension has so far received little attention, we use a computational model to investigate how a segmented tissue pattern can be maintained during convergent extension, and whether and how such a pattern may itself drive convergent extension. We use the Cellular Potts model (CPM) formalism [23, 24], which has been successfully used to model different mechanisms of convergent extension in a homogeneous tissue [12, 15], as well as several other morphogenetic processes like somitogenesis [25], ommatidia formation in *Drosophila* [26], and *Dictyostelium* culmination into a fruiting body [27]. CPM is particularly suitable for performing the type of multiscale simulations necessary to investigate convergent extension since it endows cells with an explicit size and shape, allowing for both subcellular resolution and deformation, as well as cell level properties such as adhesion and migration [28].

In this work, we show that convergent extension by itself tends to disrupt a segmented gene expression pattern that was previously formed. We demonstrate that this disruption may be counteracted by letting cells adhere preferentially to cells of the same segment type. Furthermore, we find that such segment-specific adhesion by itself can both provide the directional signal and serve as a driving force for convergent extension. When we add a simple form of directional persistence (representing inertia in the cell’s direction of movement due to the delay caused by cytoskeleton-recycling dynamics) this substantially increases the efficacy of convergent extension through segment-specific adhesion. The latter is especially true in larger and stiffer tissues, where segment-specific adhesion alone is insufficient to cause a significant tissue shape change.

## Results

### The model

For all of our simulations we used a 2D CPM model with two different cell types (red and green) which represent segments with different identities. These *in silico* cell types can either have no segment-specific adhesion -a green cell will then adhere equally strongly to a red cell as to a green cell- or have segment-specific adhesion, meaning that cells prefer to stick to cells of the same type. In both cases, cells adhere more to other cells than to the surrounding medium, such that the tissue does not fall apart into separate cells or tissue types. The medium itself has no other properties than its adhesion with cells.

In CPM, adhesion is regulated by J values, which represent the surface energy (per amount of contact surface) between cells of the same type, between cells of a different type, or a cell and medium. The CPM tries to minimize the total energy of the system, so contacts with lower J values are preferred. The strength of adhesion or repulsion between cells depends on the difference between J values, which can be conveniently represented by the surface tension (*γ*) [24]. The *γ* values are calculated from the J values as follows: $\gamma_{i,j}=J_{i,j}-\frac{J_{i,i}+J_{j,j}}{2}$, where i and j represent different cell types (m = medium, r = red, g = green). Note that *J*_*m*, *m*_ = 0. We will refer to *γ* values throughout this paper, J values are mentioned in the figure legends. A positive *γ*_*i*, *j*_ value means that cells prefer to adhere to cells of the same type, whereas negative values indicate that cells of different types prefer to mix. We will only use positive or 0 values for *γ*.

**Fig 1:**
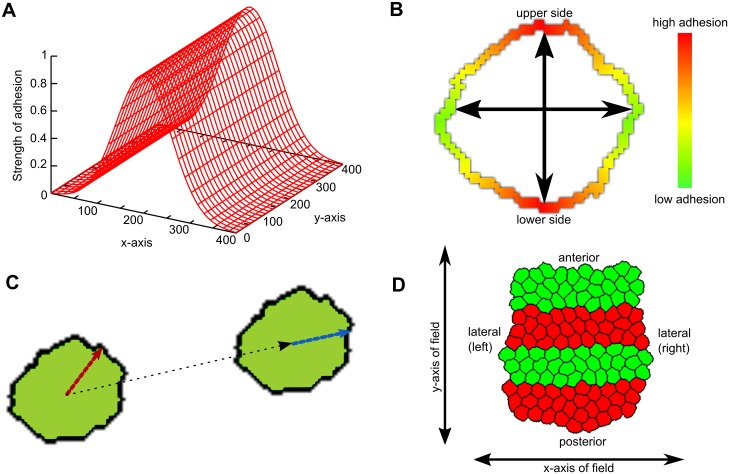
Model setup. (A) Graded adhesion. In the center of the field, adhesion between cells is higher than at the boundaries. This difference is smoothly graded according to a Gaussian distribution. (B) Axial adhesion. The parts of the membrane that are coloured red (and indicated by the vertical axis) adhere more to neighbours than the sides, which are coloured green (and indicated by the horizontal axis). The strength of adhesion is graded smoothly across the membrane, and the orientation of the axes is fixed and identical for all cells. (C) Persistence mechanism. The cell has a higher propensity to move approximately in its target direction (red vector) than the opposite direction. Every *s* Monte Carlo Steps (MCS), this vector is updated according to the actual displacement of the cell (black vector; new vector: blue). (D) Initial state of the tissue. Cells are placed closely together, so that they form a coherent tissue at the start of the simulation. The tissue is already subdivided into regular segments of identical widths and cell numbers.

For a subset of simulations, we added a so-called persistence mechanism to our model. Persistence is the tendency of cells to maintain their previous direction of movement (memory or inertia), due to the non-instantaneous turnover of the cytoskeleton [29]. When persistence is strong, cells are able to migrate rapidly in a consistent direction, as observed for example in lymphocytes and in gastrulating cells in zebrafish. We implemented persistence by giving cells a favoured (target) direction of movement. Despite this bias, the cell is not always able to move exactly in this direction due to hindrance by other cells or simply random fluctuations. Therefore, this target direction was regularly (after a fixed number of simulation steps) updated with the cell’s actual direction of displacement, representing the eventual remodelling of the cytoskeleton (Fig. 1C) [30]. A cell moving this way performs a persistent random walk.

Initially, we also tested two explicit mechanisms for convergent extension, which both used global information to direct the cells. The first mechanism, called graded adhesion, was based on the observation that mesoderm cells in zebrafish follow a gradient in cadherin activity towards the central axis ([31], reviewed in [4]). In our model, we implemented this by imposing a static gradient of cell adhesion, where the location of the cell in the field determined how strongly it adhered to neighbouring cells; cell contacts that were closer to the center of the x-axis adhered more strongly than those that were farther away (Fig. 1A). The second mechanism, called axial adhesion, was an adapted version of the mechanism presented by Zajac *et al*. [15]. This mechanism was based on the observation that intercalating cells in *Xenopus* are polarized and elongated, and the conjecture that these cells may also have a polarized distribution of adhesive molecules along their membrane. Fig. 1B shows the basic idea: the upper and lower sides of a cell (defined by the y-axis of the field) have a higher density of adhesion molecules than the left and right sides. A cell’s adhesion to a neighbouring cell is then a product of the local density of adhesion proteins on both cells, which is approximated by adjusting the J value (we don’t explicitly model adhesion proteins). We chose the axes for adhesive density such that the tissue should extend perpendicularly to the segments.

Later on, these two mechanisms were no longer applied.

For a detailed description of the implementation of all mechanisms, we refer to the Methods section.

We initiated the *in silico* tissues with a regular, segmented pattern of red and green cells (Fig. 1D). For convenience, we use the terms anterior-posterior (A-P) axis and medio-lateral (m-l) axis when we talk about the major and minor body axes of the tissue (which may have any orientation in the field). When we refer to the axes of the field (which are fixed), we simply use x-axis and y-axis. In most simulations however, the y-axis and A-P axis had the same orientation, meaning that the tissue extended in the direction of the y-axis of the field.

### Segment-specific adhesion required to maintain segments during convergent extension

**Fig 2:**
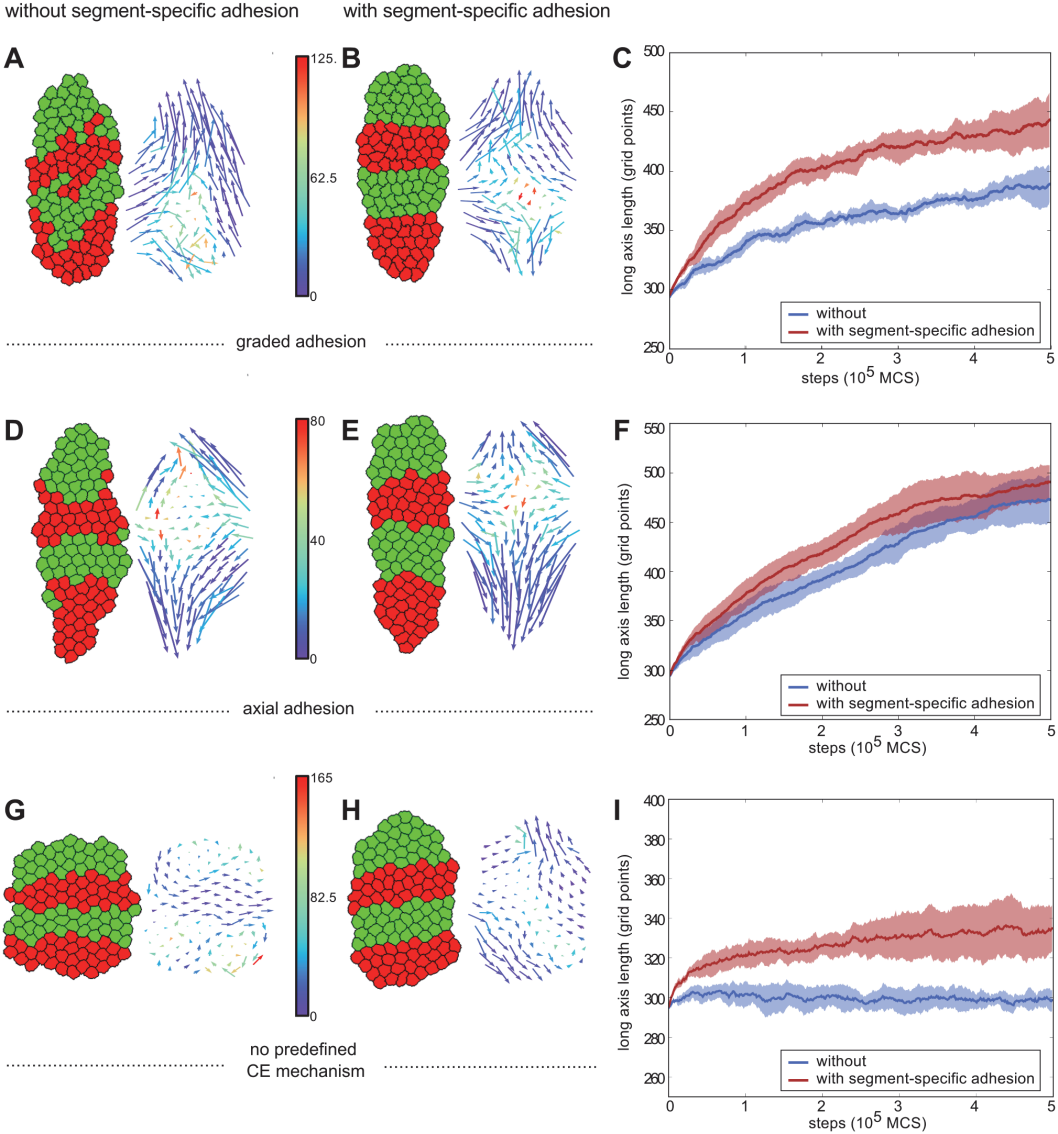
Maintenance of a segmented pattern during convergent extension requires segment-specific adhesion. Left images display tissue at the final step of the simulation (at 500,000 MCS). Right images contain the displacement vector of each cell in the simulation. The tail of a vector is located at the start position of the corresponding cell, the head at the end. The colour denotes the average angle of the vector with its neighbouring vectors. (A,B,C: row 1) Simulations with graded adhesion, strength *w* = 11. (D,E,F: row 2) Simulations with axial adhesion, strength *β* = 2. (G,H,I: row 3) Simulations without a predefined convergent extension mechanism. (A,D,G: col 1) Simulations without segment-specific adhesion, *J*_*c*, *m*_ = 10, *J*_*c*, *c*_ = 16. (B,E,H: col 2) simulations with segment-specific adhesion (*J*_*c*, *m*_ = 10, *J*_*r*, *g*_ = 16, *J*_*r*, *r*_ = 12 *γ*_*r*, *g*_ = 4) (C,F,i: col 3) Length of the long axis of the tissue as a function of simulation steps (MCS). Blue is without and red is with segment-specific adhesion. The curves are averaged over 5 runs of the model, shading indicates standard deviation.

To study the effect of convergent extension on a pre-segmented tissue pattern, we started with the incorporation of either of the two explicit global mechanisms (graded adhesion or axial adhesion) without including segment-specific adhesion or persistence. We observed for both convergent extension mechanisms that the tissues extended and narrowed, but that cells at the boundaries invaded other segments, with some losing all contact with their designated segment (graded adhesion, Fig. 2A, S1 Video; axial adhesion, Fig. 2D, S2 Video). Note that the strength of both mechanisms was relatively low in these tissues, and that the loss of segment integrity became more pronounced when the strength of the mechanisms was increased (S1 Fig.).

Next, we added a small positive surface tension between the red and green celltype (*γ*_*r*, *g*_ = 4, *γ*_*c*, *m*_ = 4), causing preferential adhesion to same-segment-type cells. This sufficed to prevent cells from leaving their segment during convergent extension (Fig. 2B and E; S3, S4 Videos). Moreover, the boundaries of the segments were much straighter. To determine whether this differential adhesion caused any additional differences, we tracked the total direction of movement of each cell over the whole course of a simulation (Fig. 2, vector plots). In all cases we see the typical pattern of convergent extension: vectors directed inwards on the lateral sides, and outwards at the anterior and posterior ends. Despite the preservation of segments when segment-specific adhesion was present, there was very little difference in the appearance of the vectors. We colour-coded the displacement vectors according to the average angle with their neighbours (Fig. 2A, B, D, E). It seems that in the presence of differential adhesion cell migratory dynamics are slightly more coherent.

The vector plots in the cases with segment-specific adhesion (Fig. 2B, E) suggest that there was a considerable amount of A-P movement, which was unexpected given that cells remained restricted to their own segment. We checked whether this restriction led to a limitation of axis extension (Fig. 2C, F). Strikingly, segment-specific adhesion did not limit axis extension, but in fact enhanced it.

### Segment-specific adhesion as a driver of convergent extension

The fact that segment-specific adhesion seemed to enhance axis extension, (Fig. 2C, F) prompted us to investigate the effect of segment-specific adhesion without any additional mechanism for convergent extension (Fig. 2G–I). Compared to tissue without segment-specific adhesion (Fig. 2G), tissue that had a small amount of segment-specific adhesion (the minimum amount needed to maintain segments in the presence of an explicit convergent extension mechanism), elongated significantly (Fig. 2H). Furthermore, convergent extension occurred without the cells or tissue having an explicit notion of their A-P axis (as opposed to the simulations in Fig. 2A–F, where the direction was imposed). This directionality now arose automatically, from the orientation of the interface between segments. The vector plot of the tissue with segment-specific adhesion (Fig. 2H) resembled the pattern generated by the graded and axial adhesion mechanisms, albeit with less extensive movement. This pattern was absent in the tissue without segment-specific adhesion (Fig. 2G). These results showed that segment-specific adhesion may not only be able to maintain segments, but could also be a driving force of convergent extension on its own.

**Fig 3:**
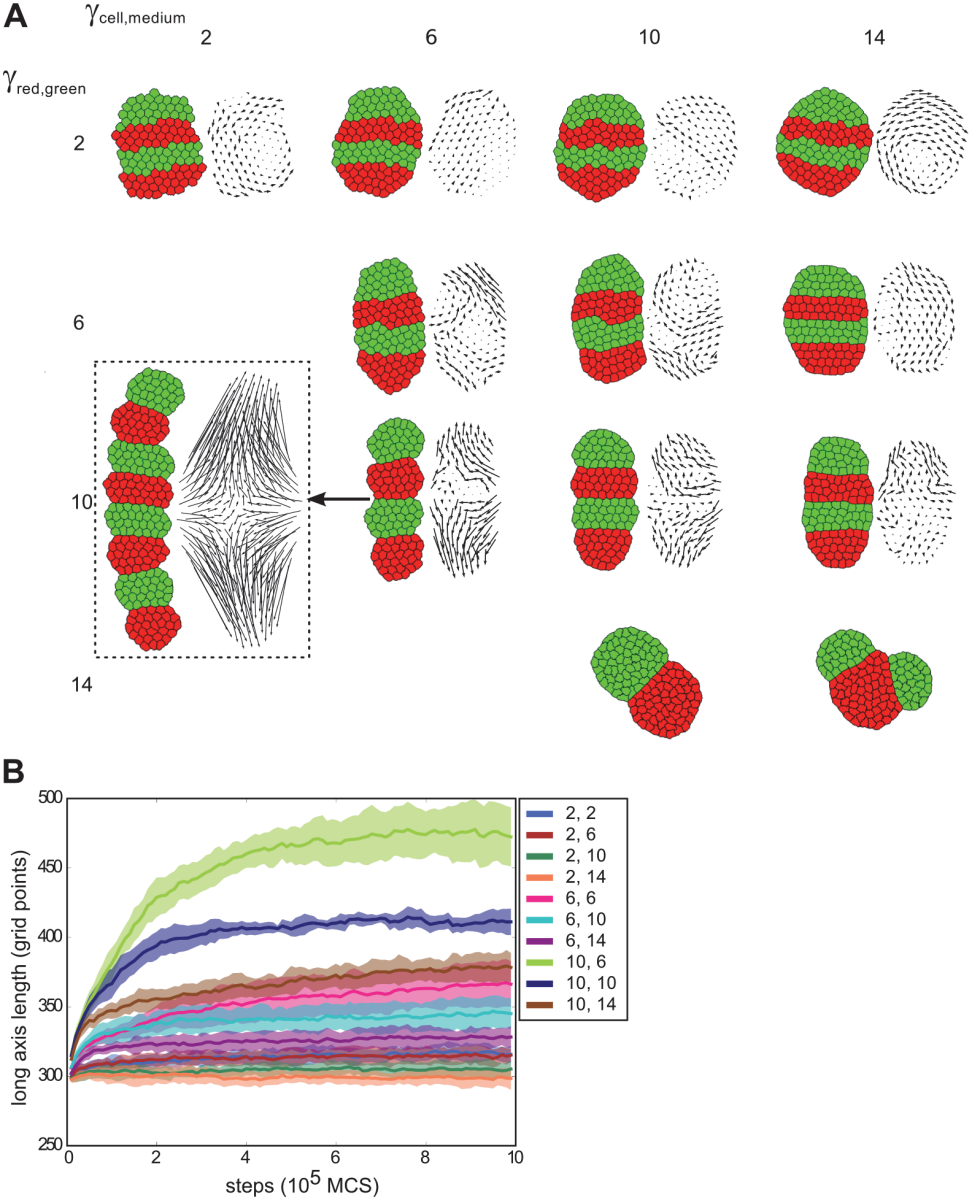
Segment-specific adhesion leads to convergent extension for a wide range of parameters. (A) Parameter space of a tissue of four segments with varying values for *γ*_*c*, *m*_ and *γ*_*r*, *g*_ (J values can be found in S1 Table). Initial segment width: 3, length: 10. For each set of parameters, 10 simulations were run over 1,000,000 MCS, representative final states are displayed. Only for the bottom two parameter sets we observed merging of segments, in resp. 9 and 8 out of 10 simulations. The dashed box shows a tissue initiated with 8 segments but with otherwise equal parameter settings to (10,6), to show the relative independence of simulation outcome from the number of segments. Vector plots were corrected for whole-tissue rotation. (B) For the same simulations, the length of the long axis of the tissue as a function of simulation steps (shading indicates standard deviation). The two parameter sets for which segments merged were not included, nor the simulation with 8 segments.

To further investigate this possibility, we varied the surface tension between red and green segments (difference in adhesion between like and unlike cells, *γ*_*r*, *g*_), and the tension of cells with the medium (*γ*_*c*, *m*_). With increasing intersegment tension, having contact surface between segments becomes less energetically favourable, creating the tendency to reduce the segment interface. This caused the segments to round up more and so become narrower and thicker, so also the entire tissue extended and narrowed more strongly for increasing (*γ*_*r*, *g*_) (Fig. 3A). Moreover, the more the tissues extended, the more the vector plots in Fig. 3A resembled the typical pattern of convergent extension.

Tissue extension is counteracted by increasing the tension with the medium, because extension and narrowing leads to a larger contact surface with the medium, which becomes less favourable with larger *γ*_*c*, *m*_. Put differently: if cells prefer not to be in contact with the medium, the tissue as a whole will remain more rounded (minimal surface with the medium) and therefore extend less. The final amount of extension therefore depended on the balance between the two opposing tensions. For the case in the parameter space with the most extreme extension (*γ*_*r*, *g*_ = 10, *γ*_*c*, *m*_ = 6), the tissue extended to about 1.5× its original length (Fig. 3B, S5 Video). When we included more and thinner segments, the tissue extended even further (to more than 2× the original length); otherwise, the results were qualitatively similar (box in Fig. 3, S2 Fig.).

Occasionally, we observed that two segments of the same celltype contacted each other and merged, thus reducing the number of segments (Fig. 3A, bottom right; S2 Fig., bottom row; S6 Video). This biologically unrealistic behaviour only occurred for very strong differential adhesion, while biologically relevant behaviour prevailed in the remaining, considerably larger part of the parameter space that we explored.

### Convergent extension by segment-specific adhesion enhanced with a persistence mechanism

So far, the *in silico* tissues with segment-specific adhesion reached their final length within about the same time scale as the explicit mechanisms. So far however, we used relatively small and loosely connected tissue. Therefore, we decided to investigate the efficacy of segment-specific adhesion in both larger and stiffer tissues.

**Fig 4:**
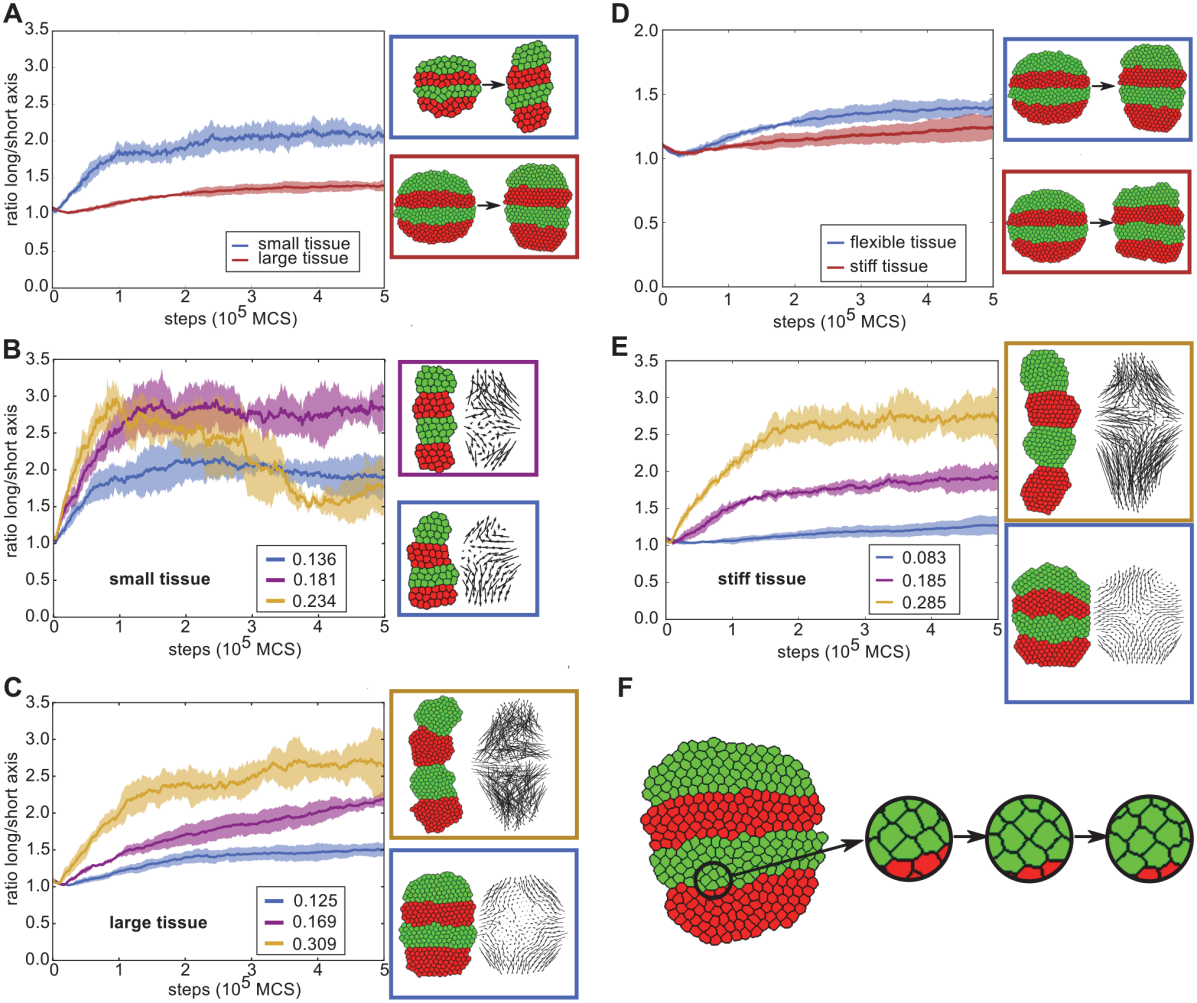
Convergent extension through segment-specific adhesion is enhanced by a persistent migration mechanism. (A) The ratio of the length of the long and short axis over simulation steps (MCS) without a persistence mechanism for a small and a large tissue (4x more cells). The large tissue (red curve) never reaches the same ratio as the small tissue. Next to the graph are examples of the state of both small and large tissue at the beginning and end of the simulation. Note that the cells in the small tissue actually are of the same size as those in the large tissue, but that the images are resized. J values: *J*_*c*, *m*_ = 12, *J*_*r*, *g*_ = 22, *J*_*r*, *r*_ = 16, *γ*_*r*, *g*_ = 6 (B,C) The long/short-axis ratio for the small tissue (B) or large tissue (C) with different cell speeds (indicated in the legends) resulting from different strengths of the persistence mechanism. Curves are averaged over 5 simulations, shading indicates standard deviation. Note that the speeds are emergent quantities measured from simulation output, and have dimension (lattice sites)/MCS (where the lattice sites have arbitrary length). Next to the graph are examples of the state at the end of the simulation (also with vector plots of cell displacement). The colour of the box indicates the parameter set the example belongs to. Vector plots were corrected for whole-tissue rotation, and in the plots for the large tissues, 60% of vectors were sampled for greater clarity. (B) Blue curve: *μ* = 0.5, *s* = 5; purple curve: *μ* = 2.0, *s* = 5; yellow curve: *μ* = 2.0, *s* = 10. (C) Blue curve: *μ* = 0.5, *s* = 5; purple curve: *μ* = 1.0, *s* = 10; yellow curve: *μ* = 1.5, *s* = 30. (D) The ratio of the length of the long and short axis for simulations with stiffer tissue, compared to the previous settings (large tissue). J values for stiffer tissue: *J*_*c*, *m*_ = 24, *J*_*r*, *g*_ = 44, *J*_*r*, *r*_ = 32, *γ*_*r*, *g*_ = 12. (E) Improvement of extension of the stiff tissue in the presence of persistence. Blue curve: *μ* = 0.5, *s* = 5; purple curve: *μ* = 2.0, *s* = 40; yellow curve: *μ* = 2.5, *s* = 40. (F) Example of T1 transition in a tissue with intermediate strength persistence. *μ* = 2.5, *s* = 20, speed 0.150, final axis extension 1.6x

In larger tissues, cells would need to travel greater distances to achieve the same degree of extension; this could potentially mean that the same process takes much longer in a larger tissue. It has indeed been suggested that if surface tension alone had to drive large changes in tissue shape, the process would take unrealistically long [32]. In Fig. 4A we compare two *in silico* tissues with the same surface tensions and the same ratio between the length and width of a segment, but one consisted of four times more cells (the number of cells in both the length and width of the segments was doubled). Because of the difference in total size, we used the ratio of the long axis over the short axis of the tissue to compare the extent of axis extension. It can be derived from first principles that for tissues with the same surface tensions and the same axis ratios at the start, the final axis ratio should be the same as well (S1 Text). As expected, the larger tissue extended at a much slower pace than the small tissue and did not reach the same axis ratio within the span of the simulation.

In the model we used so far, cells retained no memory of the direction in which they previously moved, and could change their direction of movement instantaneously. However, biological cells are to some extent persistent: due to polarization and turnover dynamics of the cytoskeleton they tend move for some time in a straight line before changing direction [29]. We hypothesized that endowing our *in silico* cells with some persistence in their movement might enhance the effectiveness of the cell motion resulting from segment-specific adhesion. Therefore, we implemented a simple persistence mechanism which has been used before in CPM for migrating lymphocytes [30] (see section “the model” and Methods for details). Note that we did not impose a tissue-level bias on the direction of persistence beforehand to favour convergent extension: the cells started each with their own random target direction.

Endowing cells with a limited tendency for persistence slightly increased the speed of cell displacement, yielding more rapid convergent extension and a more elongated tissue shape at the end of the simulation (Fig. 4B, C). In the large tissue, further increasing the level of persistence allowed the tissue to reach almost the same axis ratio as the small tissue without persistence in a comparable amount of simulation steps (Fig. 4C). The smaller tissue also gained extension speed and a larger axis ratio from increased cell speeds; however, because the tissue already extended quite rapidly, the contribution of persistence was substantially smaller (Fig. 4B). From the vector plots it can be seen that the overall cell displacement pattern still generated the typical convergent extension pattern.

Note that without differential adhesion between segments (S3 Fig.), persistent cell motion only mixed up the segmentation pattern without yielding any tissue extension. This indicates that segment-specific adhesion provided the directional signal for axis extension; aligning the initially random direction of persistent cell motion and thus allowing it to enhance tissue extension.

When the strength of the persistence mechanism was strongly increased, the probability of segments merging suddenly increased (Fig. 4B, yellow curve). Interestingly, the large tissue seemed capable of sustaining larger cell speeds before segment collapse occurred. In both cases this extreme behaviour only occurred for rather strong persistence, while in a large part of the parameter space convergent extension was significantly enhanced without the risk of tissue collapse (see also S4 Fig.).

Next, we tested the efficacy of segment-specific adhesion in stiffer, more epithelium-like tissues. For this, we doubled the J values, which reduces the amount of membrane fluctuations; as can be seen in Fig. 4D and F, the cells move considerably less and have a more distinct hexagonal shape than in the more flexible mesenchyme-like tissue we studied earlier. In these stiff tissues, segment-specific adhesion alone generates hardly any tissue extension, because the higher J values present an energy barrier to tissue shape change, much like tight junctions (Fig. 4D). However, combined with increasing levels of persistence, beyond the range of parameters used before, significant tissue extension arose (Fig. 4E). Thus, in a stiff, tightly connected tissue an active cell motility process is required to drive convergent extension, while differential adhesion still provides the directional signal. Interestingly, for intermediate persistence levels cells maintain the hexagonal shape typical of stiffer epithelial tissues and T1 transitions can be frequently observed throughout the extension process (Fig. 4F). In contrast, for the highest persistence levels tested, cells display considerable more membrane fluctuations, causing them to lose the hexagonal shape imposed by the higher tissue tension. Therefore, under these settings the tissue can no longer be considered as epithelium-like.

### Extensions to the model

**Fig 5:**
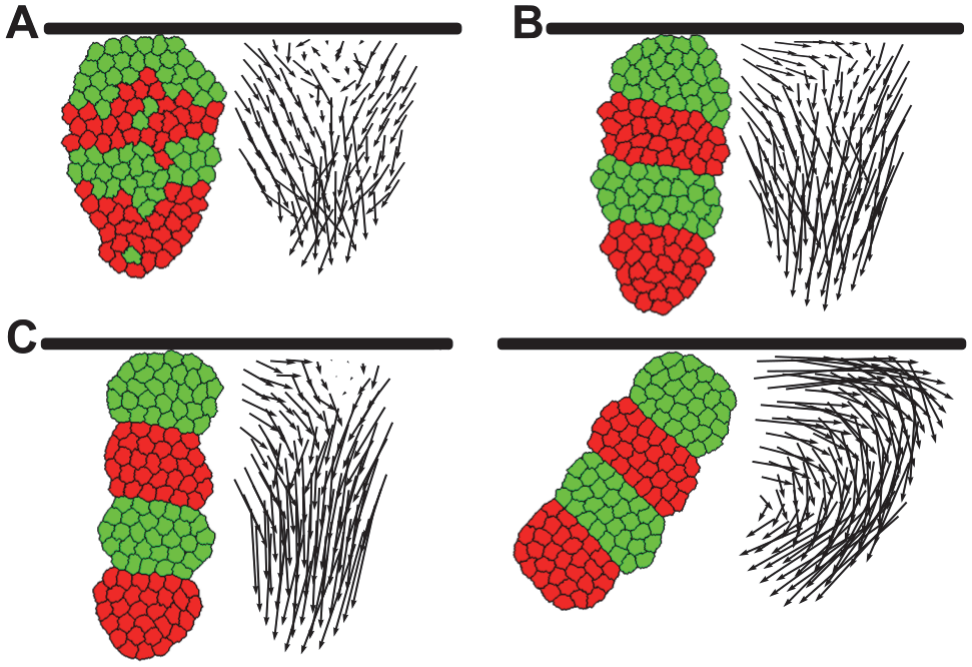
Extension to the model: a blocked anterior end does not alter results. (A,B) Simulations with graded adhesion, without (A) or with (B) segment-specific adhesion. The black bar represents the top boundary of the field (not an actual simulated object). 5 simulations were run over 500,000 MCS with parameters as in Fig. 2, representative final states are displayed. (C) Two simulations with only segment-specific adhesion, (*J*_*c*, *m*_ = 10, *J*_*r*, *g*_ = 18, *J*_*r*, *r*_ = 8 *γ*_*c*, *m*_ = 6 and *γ*_*r*, *g*_ = 10, run for 1,000,000 MCS as in Fig. 3. Note how the tissue pushed away from the boundary in the right figure; the vector field here is not corrected for tissue rotation.

The above simulations were all done with an unconstrained, fully segmented tissue. To further examine the relevance of differential adhesion as a driver of convergent extension in real-life morphogenesis, we modified our simulations in two ways corresponding to observed *in vivo* conditions: with a constrained anterior end and with a gradual instead of discretely segmented differential adhesion pattern (Fig. 5).

In many cases, the tissue undergoing convergent extension is attached on one or more sides to adjacent tissue, and is therefore restricted in its movements in that direction. For instance, in *Tribolium* the converging tissue is attached to the head, which moves very little and does not change shape. We tested the influence of such a restriction on convergent extension and cell mixing by placing the anterior end of the tissue against the border of the field, to constrain tissue movement at the anterior tissue boundary. We then applied the explicit graded adhesion mechanism (as in Fig. 2), and observed that cell mixing still occurred in the absence of segment-specific adhesion (Fig. 5A). Note that the anterior end of the tissue converged less because the tissue could not extend in the anterior direction, which becomes obvious in the vector plot of Fig. 5A where all arrows point either inward or to the posterior. This caused the tissue to become a bit ‘carrot-shaped’, which is indeed typical for extending tissues attached to non-extending tissues (see e.g., *Tribolium*). Again, segment-specific adhesion prevented mixing at the segment boundaries (Fig. 5B), and was by itself able to drive convergent extension (Fig. 5C) for larger surface tensions. Note that for strong segment-specific adhesion, the tissue tended to rotate and push away from the boundary to escape the restriction (see also vector plot), allowing it to elongate more in the same amount of simulation steps. This is an artefact of the way we modelled the restriction as only an impenetrable boundary into which no extension can occur; had the extending tissue also been attached to this boundary it would likely rotate less.

Convergent extension also occurs in non-segmented tissues. In *Xenopus* it was shown that when cells from the axial mesoderm were mixed, they quickly sorted out according to their original position on the antero-posterior axis, implicating that a position-based differentiation gradient rather than discrete segments yielded differential adhesion [20]. Strikingly, the amount of convergent extension occurring depended on the degree of sorting out that had already occurred. This suggested that differential adhesion, besides getting and keeping cells at the right position, also played an instructional role in convergent extension. Furthermore, it was found that the differential adhesion mechanism acted both upstream of and in parallel to the PCP pathway to drive convergent extension [20].

Here, we tested whether a gradient in adhesion proteins could cause correct anterior-posterior sorting and whether it could bring about convergent extension in a similar manner to the *in silico* segmented tissue. A tissue with graded expression of a single protein would not display anterior-posterior, but radial cell sorting without any convergent extension, according to both experiments and computational models [33, 34]. We therefore generated a tissue with two adhesion proteins that formed opposite gradients. This meant that a cell with a high concentration of protein A had a low concentration of protein B and vice versa (S5 Fig., see Methods for details). Cells with high A adhere more strongly to other cells with high A (and vice versa). Furthermore, cells with intermediate concentrations of both proteins adhere more strongly to each other than to cells with a high concentration of just one protein (S5 Fig., explanation in Methods).

**Fig 6:**
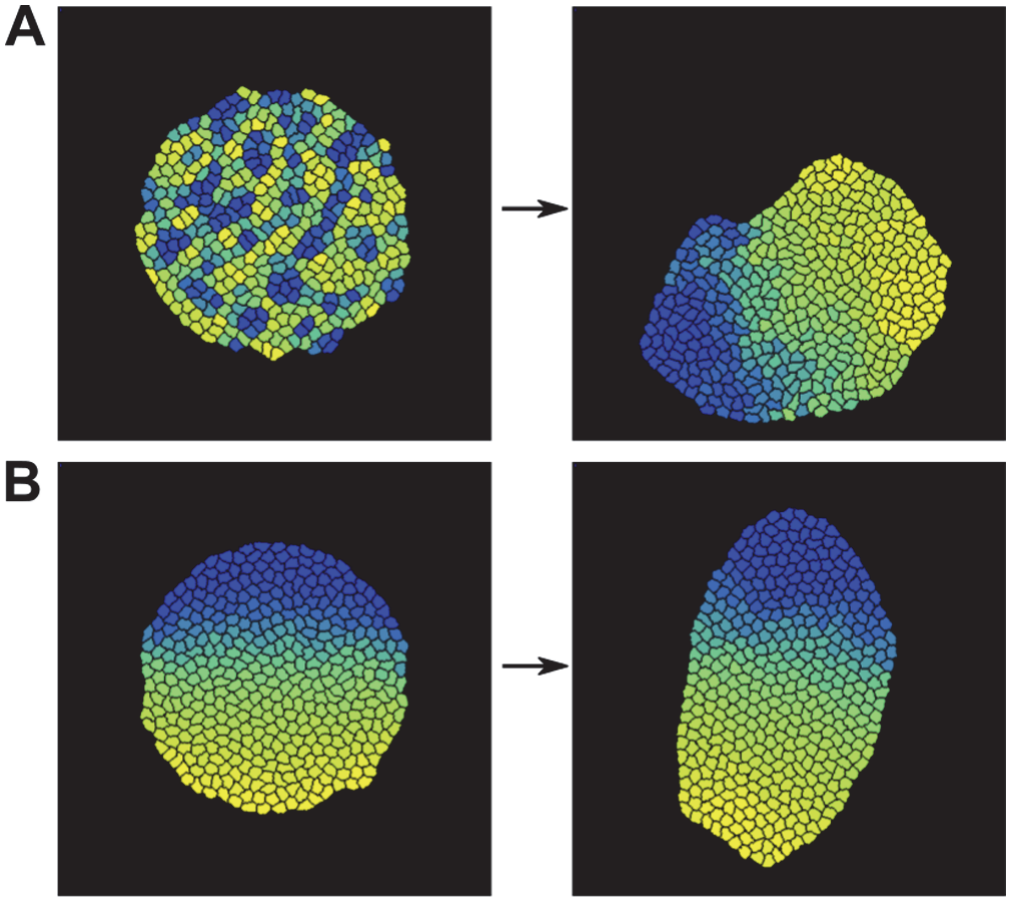
Extensions to the model: two opposing adhesion gradients lead to a-p sorting and convergent extension. (A) Random initial configuration, then run over 2,000,000 MCS. Maximum difference in adhesion strength between like and unlike cells, mm = 18. *J*_*c*, *m*_ = 15, maximum *J*_*i*, *j*_ = 28. Persistence mechanism at high strength, *μ* = 2.0, *s* = 30. (B) Simulation with two opposing gradients of adhesion proteins, sorted initial configuration (2,000,000 MCS). Persistence mechanism at low strength, *μ* = 0.5, *s* = 5.

When cells were placed randomly in the tissue (as in the experiment with mixed tissue), they sorted out with cells with similar protein concentrations clustering together. However, tissues in which cells had no persistence sorted out only partially: they became stuck in local optima where multiple clusters of similar protein concentrations were present, which was also observed for large tissues with a single protein gradient [34] (S5 Fig.). The partially sorted state was reached more quickly when the gradients of A and B concentrations were steeper, although this still did not lead to complete sorting. The tissue did sort completely when cells were endowed with high persistence, creating a rather turbulent tissue which could sort quite rapidly, with high-A cells on one end and high-B cells on the other (Fig. 6A).

When the simulation started with a tissue in which cells were already sorted, it elongated, with the extent of elongation depending on the maximum difference in adhesion (Fig. 6B, S6 Fig.). Modest persistence could enhance this process (S6 Fig.), but strong persistence reduced the extension again (see the fully sorted, but unelongated tissues in S5 Fig.). Therefore, if extension should follow after sorting of a fully mixed tissue, cell motility needs to be regulated together with the degree of sorting. However, in naturally occurring situations, AP patterning occurs prior to convergent extension, so complete mixing and hence the need for complete sorting are unlikely to occur. Rather, robustness to developmental noise will require limited sorting to optimize AP patterning, for which lower persistence levels are sufficient. Thus, our results show that besides a segmented tissue pattern, graded distributions of adhesion proteins are also capable of driving a modest form of convergent extension.

## Discussion

### Interplay of tissue patterning and convergent extension

During formation of the bilaterian body axis, cells converge and intercalate to form a tissue that is longer and narrower. Convergent extension usually occurs in tissues which have undergone prior gene expression patterning such that cells have distinct fates at different positions in the tissue. Arguably, convergent extension, which often causes extensive cell rearrangements, should be tightly regulated to prevent it from interfering with this tissue pattern. An example where this is relevant is *Tribolium*, in which convergent extension follows shortly after segmentation [17]. Paradoxically, it has been shown in both *Drosophila* and *Xenopus* that a segmented or other antero-posterior tissue pattern is required for convergent extension [20–22] suggesting that it is instructive for rather than compromised by tissue remodelling. It is therefore important to know how convergent extension may interact with a prepatterned tissue.

Here, we investigated the potential role of segment-specific adhesion in convergent extension of a fully segmented tissue. We applied two mechanisms -graded adhesion and axial adhesion- that caused convergent extension of the tissue. We demonstrated that without segment-specific adhesion, these mechanisms disturbed the segmented tissue pattern. Adding segment-specific adhesion in our model did not only preserve the segments, but also enhanced the extension of the long tissue axis. Furthermore, segment-specific adhesion by itself was sufficient for convergent extension both in unconstrained and constrained tissue, and can be combined with persistence to enhance extension in larger and stiffer tissue. Finally, we have shown that this differential-adhesion based mechanism also extends to non-segmented tissues with opposite gradients of adhesion proteins, although the amount of extension is more modest.

### Source of directionality of convergent extension

An important question concerning convergent extension is where the directional signal for the orientation of tissue extension comes from. A number of earlier models was constructed to elucidate the various mechanisms behind convergent extension through cell intercalation in different organisms [8, 10–13, 15]. Most of these predefined a direction of extension either by biasing protrusions or constrictions of the cell membrane ([8, 11, 13, 14]), or including a boundary which restricts cell motion in certain directions [10]. Only two models did not impose such a direction. In the model by Backes *et al*.([12], a positive tension between two cell types instructed the intercalation direction of forcibly elongated cells, and led to a direction of extension and narrowing which was perpendicular to that in our model. This mechanism only worked for tissues which were already quite narrow, and generated very little actual tissue extension. In the original version of the axial adhesion mechanism, constructed by Zajac *et al*. (anisotropic differential adhesion, [15]), the adhesion polarity of cells was not fixed, but rather depended on the orientation of the cell long axis (cells were forced to be elongated). In this case, the direction of tissue extension was not predefined, but emerged through alignment of the elongated cells. As a consequence, the direction of axis extension was random and differed between simulations. Finally, Shinbrot et al. [35] demonstrated that cell-cell adhesion and repulsion can generate segmented and elongated tissue patterns from random initial cell configurations. Rather than through convergent extension, the elongated and segmented patterns in their simulations form from cells condensing from a dispersed state while sorting into disks, with the tissue assuming a random orientation with respect to the field axes.

In this paper, we started out with two superimposed mechanisms for convergent extension, in which the direction of extension was also superimposed. One had an explicit gradient defining the position of the extending axis (graded adhesion), while the other imposed an internal, fixed polarity on the cells (axial adhesion), thus implicitly assuming the presence of some kind of signalling gradient. Interestingly, when segment-specific adhesion drove convergent extension alone, directionality emerged without such an imposed signalling gradient or polarity. Instead, the interface between segments provided enough information to allow the tissue to stretch in the direction perpendicular to it. The ability of segment-specific adhesion to provide the extension direction was further emphasized when we combined it with the persistence mechanism, which by itself could not produce convergent extension, but could speed up tissue extension considerably when combined with segment-specific adhesion. Therefore, to our knowledge, segment-specific adhesion is the first convergent extension mechanism which yields a predictable direction of convergent extension without imposing polarity on the cellular or tissue level.

### Mechanism of convergent extension by differential adhesion

In our model, the degree of tissue extension by segment-specific adhesion was determined by the balance between surface tension between red and green segments, and the tension of the tissue with the surrounding medium. The red-green surface tension provided the elongating force by reducing the contact surface between the segments (pulling the segment interface inward), whereas the surface tension with the medium opposed this force by making the tissue as a whole as round as possible (pulling the segment interface outward). This agrees well with findings in *Xenopus*, where the axial mesoderm needs to be enveloped in epithelium in order to extend [36]. Without the epithelial layer, the surface tension of the mesoderm with the environment is too high, resulting in a spherical tissue.

Because differential adhesion minimizes the contact area between tissues of different types, the initial ratio between segment width and length is another factor influencing the extent of convergent extension in our simulations. The smaller the initial ratio between segment width and length, the larger the contraction of the contact between segments, and the more extreme the resulting tissue elongation will be (compare Fig. 3 with ratio 3/10, to S2 Fig. with ratio 2/15). As segments are typically organized perpendicular to the length axis of the tissue, the initial segment width corresponds to the tissue width before convergent extension, while the initial segment length corresponds to the tissue length before convergent extension divided by the number of segments. The segment width length ratios used in our simulations are well within the naturally occurring ranges when considering the segment numbers and tissue widths and lengths observed in for example *Tribolium*, *Drosophila* and other arthropods.

### Limitations

We observed an apparent limit to the extent in which segment-specific adhesion can drive convergent extension. When differential adhesion tensions or persistence levels exceeded a certain threshold, the segments started to rotate and merged with other segments of the same type, thus further minimizing intersegment boundary surface. However, we suggest that this may largely be an artefact of our simplified 2D model; the risk of tissue bending may be much lower for a 3D tissue, and/or if the tissue is also embedded in other tissues (as in *Xenopus*) that restrict its movements and aid convergent extension at the same time. Furthermore, the phenomenon did not occur for most of the parameter region we tested, and we obtained strong tissue elongation within the biologically relevant region. In addition, for persistence it is reasonable to expect that once convergent extension has completed cell motility is downregulated again as part of the further progression of the development program (note that persistence is not required for maintenance of tissue extension). This termination of persistence provides an additional safeguard against segment fusion.

In the current study we have shown that in a coherent, fully presegmented tissue, segment-specific differential adhesion is a suitable candidate mechanism both for maintaining segment integrity and driving convergent extension. We did not take into account other processes that may take place at the same time as convergent extension. For instance in *Tribolium* and other short-germ insects, the segments are laid down sequentially instead of simultaneously, from a growth zone where cell division provides a steady source of undifferentiated tissue. It appears that in this case, convergent extension occurs shortly after a new segment is laid down [16, 17]. Based on preliminary results, we expect that segment-specific adhesion will also suffice to drive convergent extension during sequential segmentation, but given the complexity of the growth zone and segment-definition dynamics, it is beyond the scope of this article to investigate this.

Furthermore, we assumed for the sake of simplicity that a cell’s adhesion is a fixed property. However, we recognize that this may not always be the case, for instance when cells change the concentration of adhesion molecules on their membrane in response to interactions with other cells that possess different (concentrations of) adhesion molecules (see [33]). This may influence the ability of differential adhesion to drive convergent extension.

### Comparison to experiments

We found that for loosely connected, mesenchyme-like tissues differential adhesion alone or combined with a limited persistence of motion can drive convergent extension. As such, we expect differential adhesion to contribute to axial extension in organisms such as *Xenopus* in which an antero-posterior pattern is present, and which indeed served as one of the inspirational starting points for this study. Possibly this mechanism also plays a role in short-germ insects such as *Tribolium*, which undergoes convergent extension simultaneously with segmentation, if the tissue emanating from the growth zone is indeed flexible enough.

For stiffer tissues we found that in order to obtain substantial tissue extension, segment-specific adhesion needs to be combined with a significant level of persistent cell motion. Notably, the persistence alone would not produce any convergent extension, but requires differential adhesion to instruct and coordinate cell movement. Furthermore, the strength of persistence required for proper extension was so low that inspection by eye would most likely not reveal the presence of this mechanism in *in vivo* tissues, as the cell displacement is similar to that of tissues where cells are not persistent. Persistence strong enough to be visible led to turbulence and segment merging, and would require other, more global directional cues than segment-specific adhesion to yield convergent extension.

Although clearly not a one-to-one match, persistence bears intriguing similarities to the case of the *Drosophila* germband. Here, parasegmental actomyosin barriers prevent intersegmental cell mixing [37, 38], while the segments also serve as a directional signal for planar cell polarity, which subsequently instructs the anisotropically directed actomyosin contractions that drive the T1 transitions underlying convergent extension [7–8, 22]. The similarities reside in the fact that the segmental pattern instructs the direction of cell movement, and that cell movement requires active cytoskeletal remodeling. It remains to be established whether segment-specific adhesion can act in combination with and thereby enhance the mechanisms observed in *Drosophila*, or whether it may act as an alternative strategy deployed in other organisms.

Unfortunately, the similarities between the differential adhesion mechanism and the *Drosophila* type mechanism make the design of an experimental setup to discriminate against these two possibilities highly non-trivial. As an example, if we experimentally disrupt genetic factors regulating segmentation these will not only hamper segment-specific adhesion, but also the aforementioned planar polarity such as occurs in *Drosophila*. As a consequence results would be inconclusive. Likewise, active cytoskeletal dynamics are involved both in convergent extension driven by the combination of differential adhesion and persistence and in planar-polarized junctional tension driven convergent extension. Thus, failure of convergent extension upon actomyosin disturbance will again be inconclusive. Similarly, although one could try to experimentally increase the adhesiveness of the whole tissue by ubiquitously expressing e.g. N-cadherin; this would certainly hinder convergent extension via segment-specific adhesion, but unfortunately is likely to also hinder other convergent extension mechanisms by increasing the energy required to break the bonds between cells. This problem of distinguishing between the two mechanisms is further aggravated by the fact that the mechanisms may be likely to work in combination. One experiment that may allow for a distinction between the two convergent extension mechanisms is to apply pulling forces on the tissue in the direction parallel and perpendicular to the segmentation pattern. If less force is required to tear the tissue along segment boundaries than to tear it in the perpendicular direction this is a strong indicator that differential adhesion is involved. Still, this does not allow one to establish the importance of this differential adhesion for convergent extension.

### Conclusion

In summary, we have shown that differential adhesion is sufficient to drive convergent extension in presegmented tissues, and represents a convergent extension mechanism not requiring any directional signal. While the investigated convergent extension mechanism may not be universal, in segmented tissues the presence of segmental boundaries is likely to contribute to convergent extension, either via differential adhesion or via alternative mechanisms such as actomyosin bands or planar cell polarity. Likewise, while not all tissue is segmented, anterior posterior patterning may also allow for differential adhesion-based convergent extension. In the current study we focused on the role of a fully presegmented tissue pattern in driving convergent extension. However, in many cases segmentation and convergent extension occur simultaneously. Therefore, in future work we aim to investigate the dynamic interplay between sequential segmentation and convergent extension. Considering such bidirectional feedback between patterning and morphogenesis may bring to light important principles of coordinating growth and patterning.

## Methods

### Model setup

#### CPM model formalism

We consider convergent extension of monolayer tissues, and therefore we use a simple 2D Cellular Potts Model for all our simulations. In the CPM model formalism, cells consist of multiple lattice sites with 2D coordinates i and j and have a cell type *τ* and identification number *σ*. The lattice (“field”) is updated using the Monte Carlo algorithm. For each Monte Carlo step (MCS), lattice sites are drawn randomly, as many times as there are lattice cells. For each site belonging to the boundary of a cell, a random neighbour is selected which may copy its identity into this lattice site. The probability of this event is calculated with the Hamiltonian, which depends on the change in cell surface energy and cell volume that would be caused by the potential copy event. The surface energy of lattice sites at the cell boundary, *J*_*τ*(*σ*_*ij*_), *τ*(*σ*_*i*^′^*j*^′^_)_, depends on the type of the cell (*τ*(*σ*_(*ij*)_)) and that of the neighbour that the lattice site contacts (*τ*(*σ*_*i*^′^*j*^′^_)). Cells are assumed to minimize their surface energy while at the same time trying to maintain their volume (or in 2D, area) at a target value *A*_*σ*_. The Hamiltonian is then as follows:

$$H=\sum_{ij}\sum_{i^{\prime }j^{\prime }}J_{\tau (\sigma_{ij}),\tau (\sigma_{i^{\prime }j^{\prime }})}(1-\delta_{\sigma_{ij,}\sigma_{i^{\prime }j^{\prime }}})+\sum_{\sigma }\lambda_{a}{\left(a_{\sigma }-A_{\sigma }\right)}^{2}$$

The first term represents the sum of all surface energies J, where *δ* is the Kronecker delta (0 if *σ*_*ij*_ and *σ*_*i*^′^*j*^′^_ are different, and 1 if they are equal). *σ*_*i*^′^*j*^′^_ sums over all 8 neighbouring sites in the 3 × 3 neighbourhood. The second term serves to keep the actual area *a* of a cell close to the target area *A*, where *λ*_*a*_ is the resistance of cells against volume changes. The probability that a neighbouring site extends into the lattice site under consideration is 1 if Δ*H* < 0, and *e* ^−(Δ*H*)/*T*^ otherwise, where Δ*H* is the change in the Hamiltonian due to the considered modification, and T is the simulation temperature, determining the membrane fluctuation amplitude of cells. The model was implemented using the C++ programming language.

#### J values and adhesion

As mentioned before, the J values represent the surface energies at the interface of cells and their surroundings. The higher the J value, the less favourable the interaction, and therefore a pixel copy which reduces such an interface will be rather likely. Therefore, mechanisms which increase adhesion reduce the J value of that cell-cell interaction. J values act in a relative manner. If the J value of a cell-medium interaction is lower than that of a cell-cell interaction, cells will disperse through the medium rather than form a coherent tissue. Likewise, if the J value between red and green cells is higher than both the J value of red-red and green-green interactions, red and green cells will form a chequerboard pattern (see [24] for more details). The behaviour of cells under a certain set of J values can be more easily seen from the surface tensions, which are calculated from the J values as follows:

$$\gamma_{i,j}=J_{i,j}-\frac{J_{i,i}+J_{j,j}}{2}$$

where i and j represent different cell types. Now, a positive *γ* value for the interaction between red and green cells keeps them separated (while a negative one causes mixing), and the same is true for the *γ* value of cell and medium interactions.

#### Simulation initialisation

Cells are initiated as blocks of 5 × 5 lattice sites, put closely together in a grid-like manner in the center of the field. At this point, we already divide the *in silico* tissue into regular segments of alternating cell types. In the simulations of Fig. 2 and Fig. 3, each segment is 3 cells thick and 10 cells wide upon initialization. In the simulations for Fig. 4, the segments were each 2 × 10 cells for the small tissues, and 4 × 20 for the large tissues.

### Explicit convergent extension mechanisms

Two alternative mechanisms for convergent extension were used: graded adhesion and axial adhesion. To implement such a mechanism, Δ*H* is modified for certain lattice sites to establish a bias in copy probability.

#### Graded adhesion

For the graded adhesion mechanism, the J value of two adjacent cells is modified depending on their location within the field. We defined a Gaussian function with a maximum at the center of the field in the x-direction (Fig. 1A), but homogeneous in the y-direction.

$$J^{\prime }=J-w*e^{-\frac{{\left(x-b\right)}^{2}}{2*c^{2}}}$$

where w is the maximum amplitude of the modification, b is the center of the x-axis of the field, and c is the standard deviation of the Gaussian. As a consequence, J values become gradually lower towards the center of the field.

#### Axial adhesion

For the axial adhesion mechanism, cells have an increased adhesion on the upper and lower faces of the cell (Fig. 1B). This was incorporated using the following modification of the J values:

$$J^{\prime }=J-\beta^{2}*|sin(\alpha )|*|sin(\alpha^{\prime })|$$

where *β* represents the maximum reduction of surface energy because of the mechanism, and *α* and *α* ^′^ are angles with the horizontal (x-) axis. *α* is the angle of the vector pointing from the center of cell *σ*_*ij*_ to the membrane segment where the copy takes place, and *α* ^′^ is the angle of the vector in the neighbouring cell (*σ*_*i*^′^*j*^′^_). By taking the sine, we ensure that the modification of *J* is highest for membrane segments both at the top and bottom of the cell (so with vectors along the y-axis), correcting with the absolute value for the fact that angles larger than *π* yield negative sines. See Zajac *et al*. [15] for the original version.

#### Persistent motion

Persistent motion is implemented by enhancing the probability of extension in the direction of previous movement. In practice, this means that cells have a target direction, and extensions that have a small angle with this direction occur with a higher probability than extensions with a large angle to the target. The target direction is updated every *s* MCS to the direction of the actual previous displacement of the cell. Persistence was incorporated by extending Δ*H* as follows:

$$\Delta H^{\prime }=\Delta H-\mu \cos(\zeta )$$

where *μ* is the strength of persistence, and *ζ* is the angle between the target direction and the displacement vector under consideration (i.e., the vector given by the mean position of the cell and the coordinates of the position to be modified). Both larger *μ* and larger update times contribute to higher persistence (time during which the cell moves in a more or less straight line) and larger cell speed. See also Fig. 1C and [30].

#### Opposing adhesion protein gradients

Cells have two adhesion proteins A and B, whose concentrations together add up to 1.0 (arbitrary units). So *A* = 1.0 − *B*. For the sorting simulations, cells are assigned a random concentration of B between 0 and 1. Subsequently, their concentration for A follows from *A* = 1.0 − *B*. In the convergent extension simulations, where the cells are already sorted, the concentration of B is increased stepwise from 0 to 1 in every subsequent row. The protein concentrations are translated to a J value between two cells i and j as follows:

$$J_{i,j}=J_{i,j}-mm\;*\;(min(A_{i},A_{j})+min(B_{i},B_{j}))$$

where *A*_*i*_ is the concentration in cell i, min compares the concentrations of cell i and j and takes the minimum, and mm is the maximum reduction in J value (when cells have identical protein concentrations). MM therefore determines the ‘steepness’ of the adhesion gradients. The cell with the lowest concentration of a protein dictates the amount of adhesion between two cells via that protein, so a cell with high A still adheres poorly to a cell with low A. The two adhesion proteins do work additively: having a bit of both leads to more adhesion than just having a bit of one; therefore, cells with intermediate concentration adhere more strongly to each other.

### Model analysis

#### Cell displacement vectors

The mean position of each cell is registered every 1000 MCS. The position at time 5000 (to allow for an initialization period) and the final time point are used to determine the overall displacement vector (using Python scripts). The vector plots are corrected for whole-tissue rotation with respect to the y-axis of the field. For this, the rotation of the long axis of the tissue with respect to the y-axis is registered throughout the simulation.

#### Correlation of neighbouring cell displacements

The displacement vectors of Fig. 2 and S1 Fig. have been coloured according to their average angle with the neighbouring vectors. A neighbour of a vector has a starting point which lies within a neighbourhood of size 30 (field pixels) around the starting point of the vector. The colour bar is scaled relative to the largest average angle in one of the two simulations to be compared (see Fig. 2). The colour of the vector is relative to the largest value

#### Emergent cell displacement speed

The mean positions are also used to calculate cell speeds, which is both an average over the variable speed of a cell and an average between cells. We used customized Perl scripts for the calculations.

### Model parameters

The current study serves as a proof of principle, illustrating how convergent extension may disrupt pre-existing tissue patterns, while these pre-existing tissue patterns may also drive convergent extension through differential adhesion. Because of the conceptual nature of our model, we do not aim to quantitatively fit convergent extension dynamics in a particular model organism. However, if this where to be the case, model parameters could be adjusted to obtain cell movement speeds and trajectories matching experimental data. In contrast, in the current study we aim to illustrate that differential adhesion either alone or combined with persistence of motion, represents a feasible new mechanism for convergent extension. As such, we aimed to ensure that differential adhesion driven convergent extension occurs for a wide range of parameters, making it a plausible mechanism in broad range of contexts. For persistence, parameter scaling was done internally: we matched persistence tendencies to membrane fluctuations and overall tissue deformation so that we remained in the domain of biologically realistic behaviour, avoiding the merging of segments or of turbulent tissue dynamics. As a consequence, we applied considerably lower persistence tendencies than in the study by Beltman et al [30], where it was used to simulate migrating lymphocyte dynamics. Note the limited persistence tendencies applied in our studies significantly altered quantitative model behaviour, as shown in Fig. 4. Default parameter values are shown in Table 1.

**Table 1:** Default parameter values.

**parameter**	**value**	**comments**
*common parameters*		
field size	400×400–800×800 (lattice sites)	size varied to accommodate tissue even when extended
duration	5 * 10^5^ (MCS)	
initialization period	500 (MCS)	
simulation temperature (T)	15	
neighbourhood order	2	
target cell area	500 or 200 (lattice sites)	smaller cell size used for all simulations with persistence (for computational efficiency)
*λ*_*a*_	1.	
*J*_*c*, *m*_	10–22	ranges used in all simulations except in those with stiff tissue (Fig. 4D–F)
*J*_*r*, *g*_	12–18	
*J*_*r*, *r*_	4–18	
*mechanism-specific parameters*		
w	11	
c	7	
*β*	2.0	
*μ*	0.5–2.0	
*s*	5–30 (MCS)	
*mm*	12–24	

Where applicable, parameter dimensions are indicated behind the value between parentheses.

## Supporting Information

S1 Fig Strong graded adhesion and axial adhesion lead to more elongation and more mixing at the segment boundaries. Left images display tissue at the final step of the simulation (at 500,000 MCS). Right images contain the displacement vectors of each cell in the simulation. The tail is located at the start position of the cell, the head at the end. (A,B,C: row 1) Simulations with graded adhesion, strength *w* = 12. (D,E,F: row 2) Simulations with axial adhesion, strength *β* = 2.66. (A,D) Simulations without segment-specific adhesion. (B,E) Simulations with segment-specific adhesion (*γ*_*r*, *g*_ = 4). (C,F) Length of the long axis of the tissue as a function of simulation steps. Blue is without and red is with segment-specific adhesion. The curves are averaged over 5 runs of the model, shading indicates standard deviation. Note that the added effect of segment-specific adhesion on axis extension is smaller here than when the convergent extension mechanisms are weaker (compare to Fig. 2 in main article).
(PDF)

S2 Fig Segment-specific adhesion leads to greater extension with more and narrower segments. Parameter space of a tissue of eight segments with varying values for *γ*_*c*, *m*_ and *γ*_*r*, *g*_, same as Fig. 2 (See S1 Table for J values). Initial segment width:2, length:15. cells, For each set of parameters, 10 simulations were run over 100,000 MCS, representative final states are displayed. In the following simulations we observed merging of segments [(row, col), # out of 10 sims]: (10,10) 1; (10,14) 2;(14,10) 10; (14,14) 10. Vector plots were corrected for whole-tissue rotation.
(PDF)

S3 Fig Without segment-specific adhesion, persistence does not lead to convergent extension. On the left: a tissue without segment-specific adhesion, and no persistence mechanism. On the right: a tissue without segment-specific adhesion, having a persistence mechanism with *μ* = 2.0, *s* = 10, leading to an average cell speed of 0.181 (lattice sites/MCS). J values are *J*_*c*, *m*_ = 12, *J*_*c*, *c*_ = 18
(PDF)

S4 Fig Influence of persistence on cell and tissue dynamics. (A) Influence of persistence on tissue elongation. Results are shown for simulations with varying persistence parameters (*μ* = 0 − 2.0, *s* = 5 − 30) with the long/short axis ratio at the end of the simulation (duration 5*x*10^5^ MCS) plotted against the measured average cell speed of a single isolated cell with those parameters(lattice sites/MCS). J values are *J*_*c*, *m*_ = 12, *J*_*r*, *g*_ = 22, *J*_*r*, *r*_ = 16, *γ*_*r*, *g*_ = 6. (B) For a subset of the persistence levels in A, cell tracks from 5 random cells part of the same extending tissue are shown (1 of the 5 simulated tissues shown in A; parameters correspond to the following cell speeds (single, tissue): (0.60,0.117), (0.69,0.137), (0.80,0.169), (0.91,0.211), (1.01,0.309), (1.10,0.343), (1.29, 0.501)). The tracks are measured over 100 000 MCS, with the start of each track shifted to the center. Different tracks are depicted with different colors. (C) For the same subset of persistence levels as shown in B, cell tracks of single-cell simulations (100 000 MCS) are shown. The tracks become lighter with age to indicate directionality. The right-most cell track is of a single cell with strong, lymphocyte-like persistence (*μ* = 16, *s* = 8), parameters are as in Vroomans *et al*., PLoS Comp. Biol. 2011. Note the qualitative difference: the cell turns less often, and has more straight stretches (field size 2000×2000). N.B. Track does not become lighter with age.
(PDF)

S5 Fig Opposing adhesion gradients lead to (partial) sorting out of tissue. The graph shows how the adhesion proteins are distributed in the tissue, and the corresponding cell colour. The images show the tissue at the end of the simulation (2,000,000 MCS) for varying strengths of the maximum adhesion difference mm, without or with persistence (parameters *μ*: 2.0 and *s*: 30–40). *J*_*c*, *m*_ = 15, maximum *J*_*i*, *j*_ = 28.
(PDF)

S6 Fig Opposing adhesion gradients lead to modest tissue extension. The graph plots the length of the long axis of the tissue over simulation steps for varying values of the maximum adhesion strength (mm: 12, 18, 24), and without or with persistence mechanism (parameters *μ*: 1.0 and *s*: 10).*J*_*c*, *m*_ = 15, maximum *J*_*i*, *j*_ = 28.
(PDF)

S1 Video Simulation with graded adhesion, strength *w* = 11, and no segment-specific adhesion. Duration is 500 000 MCS.
(MP4)

S2 Video Simulation with axial adhesion, strength *β* = 2, and no segment-specific adhesion. Duration is 500 000 MCS.
(MP4)

S3 Video Simulation with graded adhesion, strength *w* = 11, and segment-specific adhesion (*γ*_*r*, *g*_ = 4). Duration is 500 000 MCS.
(MP4)

S4 Video Simulation with axial adhesion, strength *β* = 2, and segment-specific adhesion (*γ*_*r*, *g*_ = 4). Duration is 500 000 MCS.
(MP4)

S5 Video Simulation with segment-specific adhesion, (*γ*_*c*, *m*_ = 6, *γ*_*r*, *g*_ = 10). Duration is 1 000 000 MCS.
(MP4)

S6 Video Simulation with segment-specific adhesion (segment collapse), (*γ*_*c*, *m*_ = 10, *γ*_*r*, *g*_ = 14). Duration is 1 000 000 MCS.
(MP4)

S1 Table This table contains the J values of the parameter space of Fig. 3 and S2 Fig. (PDF)

S1 Text Additional information. This document explains how the equilibrium contact length between two segments can be calculated from the area of the segments and the surface tensions.
(PDF)
