# Supplementary figures and images for: Segment-Specific Adhesion as a Driver of Convergent Extension
Source: PLoS Comput Biol. 2015 Feb 23;11(2):e1004092. doi: 10.1371/journal.pcbi.1004092 (PMC4338282; doi:10.1371/journal.pcbi.1004092)

without segment-specific adhesion      with segment-specific adhesion

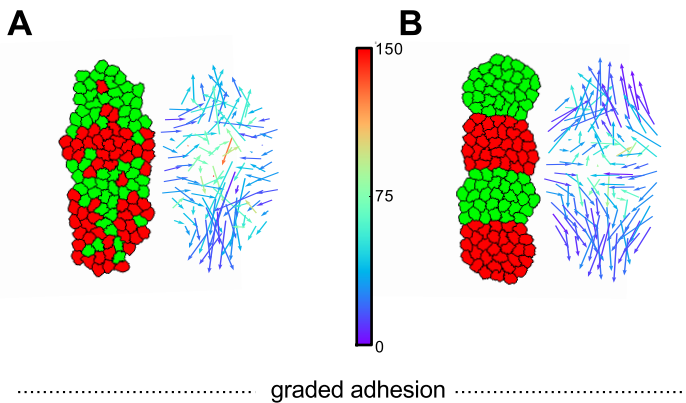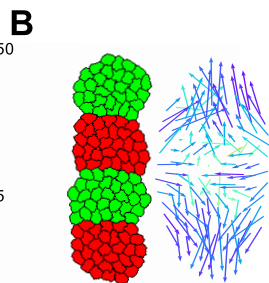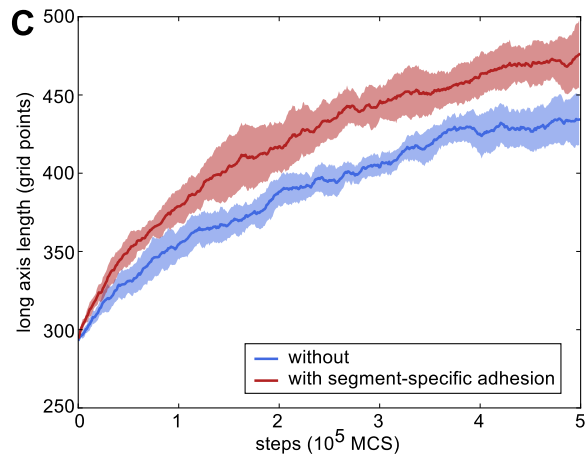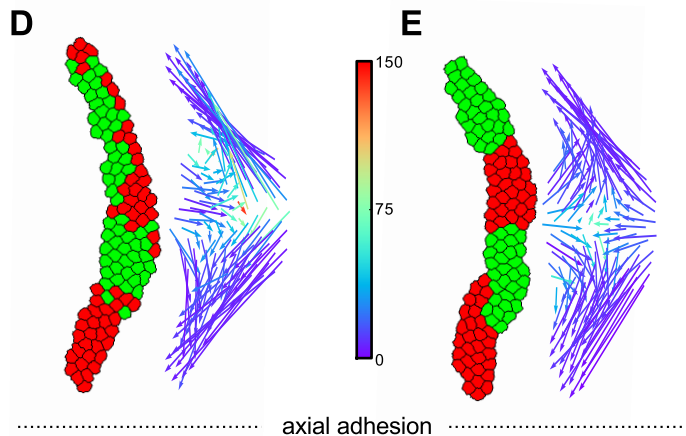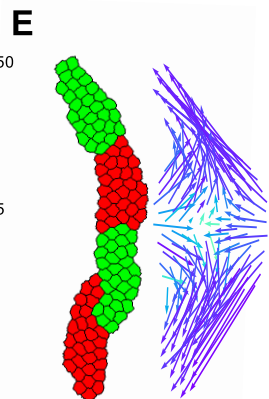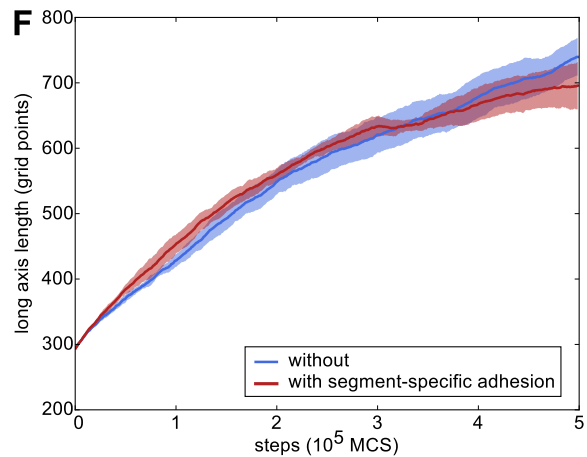

Supplement: S1 Fig — Left images display tissue at the final step of the simulation (at 500,000 MCS). Right images contain the displacement vectors of each cell in the simulation. The tail is located at the start position of the cell, the head at the end. (A,B,C: row 1) Simulations with graded adhesion, strength w = 12. (D,E,F: row 2) Simulations with axial adhesion, strength β = 2.66. (A,D) Simulations without segment-specific adhesion. (B,E) Simulations with segment-specific adhesion (γ r, g = 4). (C,F) Length of the long axis of the tissue as a function of simulation steps. Blue is without and red is with segment-specific adhesion. The curves are averaged over 5 runs of the model, shading indicates standard deviation. Note that the added effect of segment-specific adhesion on axis extension is smaller here than when the convergent extension mechanisms are weaker (compare to Fig. 2 in main article). (PDF) [file pcbi.1004092.s001.pdf]

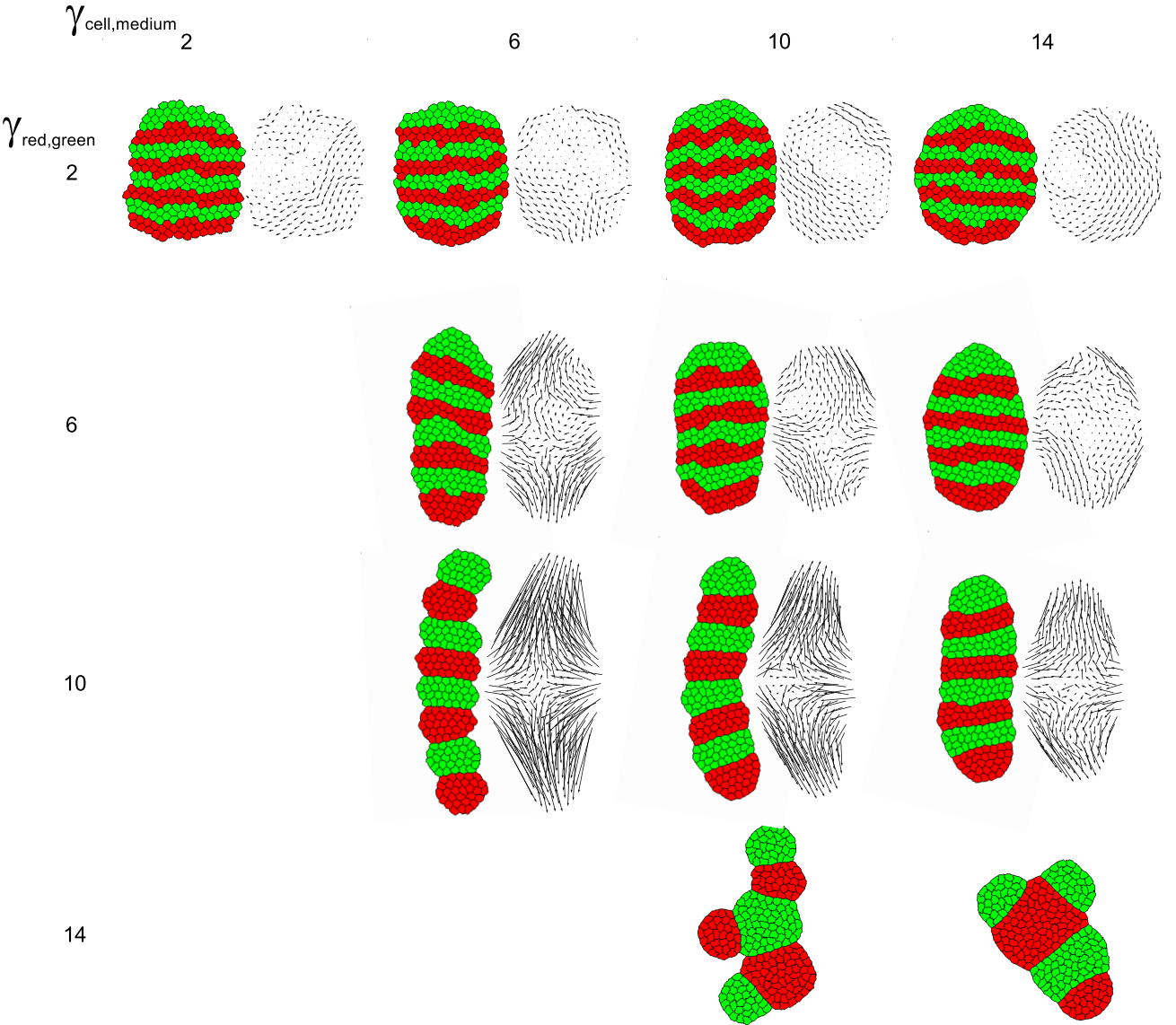

Supplement: S2 Fig — Parameter space of a tissue of eight segments with varying values for γ c, m and γ r, g, same as Fig. 2 (See S1 Table for J values). Initial segment width:2, length:15. cells, For each set of parameters, 10 simulations were run over 100,000 MCS, representative final states are displayed. In the following simulations we observed merging of segments [(row, col), # out of 10 sims]: (10,10) 1; (10,14) 2;(14,10) 10; (14,14) 10. Vector plots were corrected for whole-tissue rotation. (PDF) [file pcbi.1004092.s002.pdf]

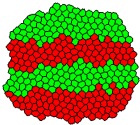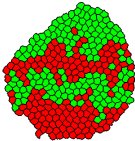

Supplement: S3 Fig — On the left: a tissue without segment-specific adhesion, and no persistence mechanism. On the right: a tissue without segment-specific adhesion, having a persistence mechanism with μ = 2.0, s = 10, leading to an average cell speed of 0.181 (lattice sites/MCS). J values are J c, m = 12, J c, c = 18 (PDF) [file pcbi.1004092.s003.pdf]

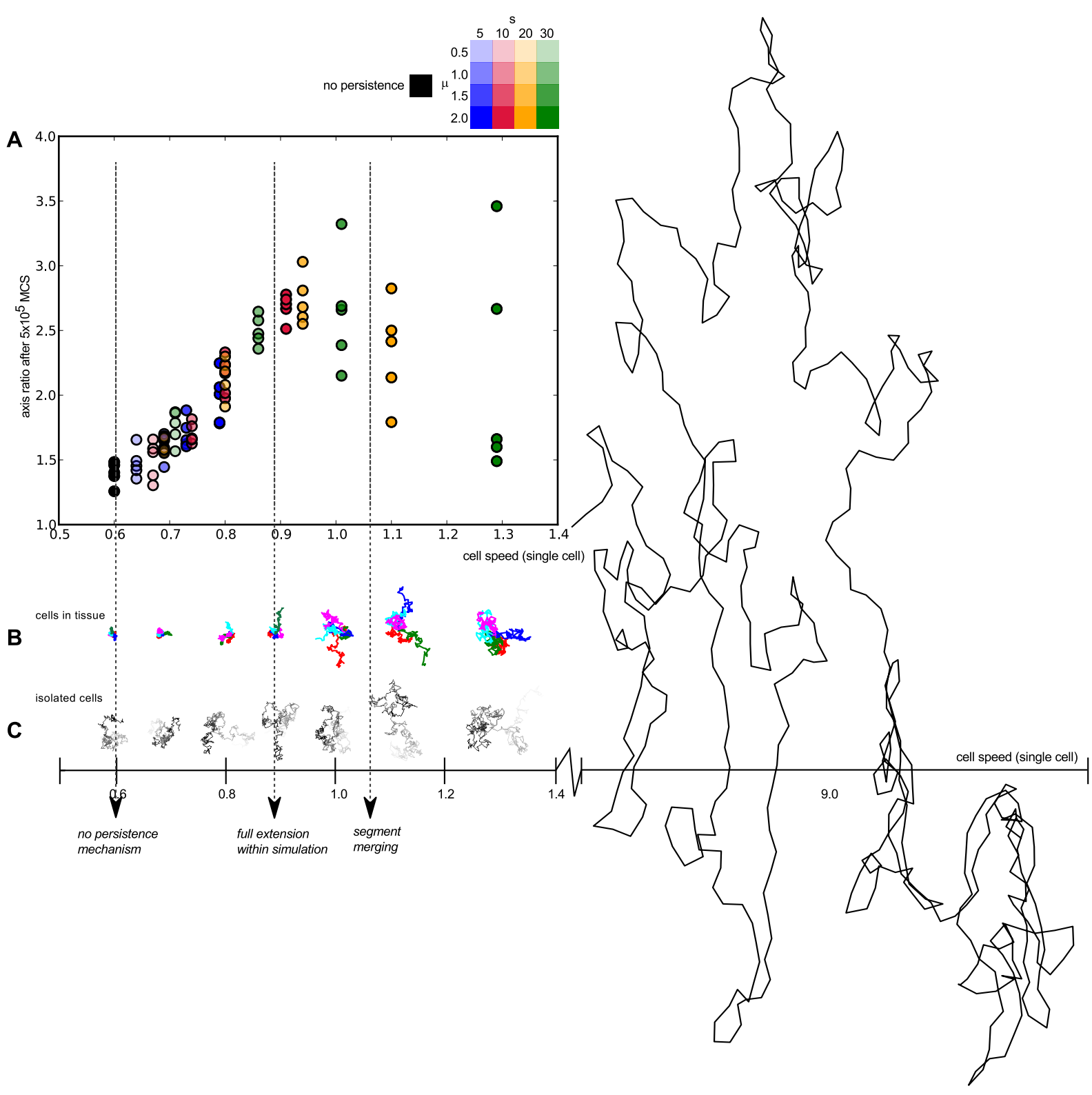

Supplement: S4 Fig — (A) Influence of persistence on tissue elongation. Results are shown for simulations with varying persistence parameters (μ = 0 − 2.0, s = 5 − 30) with the long/short axis ratio at the end of the simulation (duration 5x10^5^ MCS) plotted against the measured average cell speed of a single isolated cell with those parameters(lattice sites/MCS). J values are J c, m = 12, J r, g = 22, J r, r = 16, γ r, g = 6. (B) For a subset of the persistence levels in A, cell tracks from 5 random cells part of the same extending tissue are shown (1 of the 5 simulated tissues shown in A; parameters correspond to the following cell speeds (single, tissue): (0.60,0.117), (0.69,0.137), (0.80,0.169), (0.91,0.211), (1.01,0.309), (1.10,0.343), (1.29, 0.501)). The tracks are measured over 100 000 MCS, with the start of each track shifted to the center. Different tracks are depicted with different colors. (C) For the same subset of persistence levels as shown in B, cell tracks of single-cell simulations (100 000 MCS) are shown. The tracks become lighter with age to indicate directionality. The right-most cell track is of a single cell with strong, lymphocyte-like persistence (μ = 16, s = 8), parameters are as in Vroomans et al., PLoS Comp. Biol. 2011. Note the qualitative difference: the cell turns less often, and has more straight stretches (field size 2000×2000). N.B. Track does not become lighter with age. (PDF) [file pcbi.1004092.s004.pdf]

Cell colour

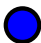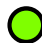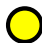

protein concentration

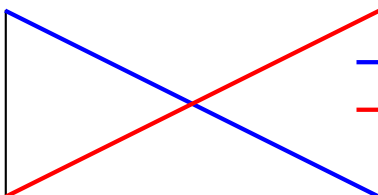

— Protein A

— Protein B

start

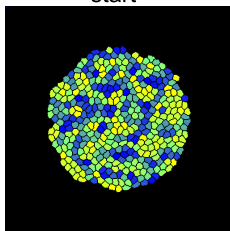

mm=12

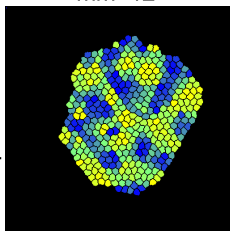

mm=18

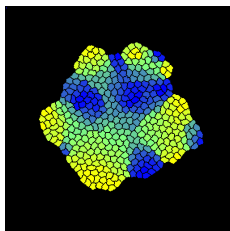

mm=24

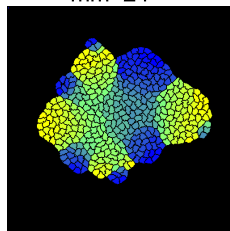

no persistence

$\mu=2.0, s=30$

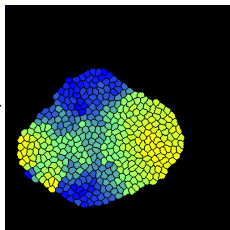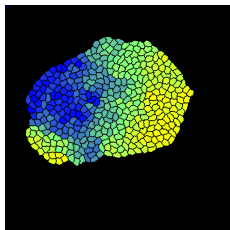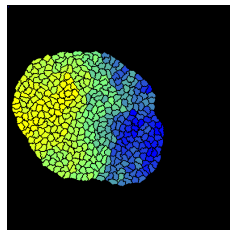

$\mu=2.0, s=40$

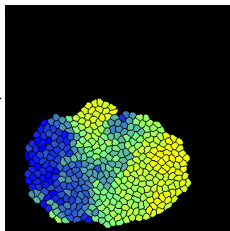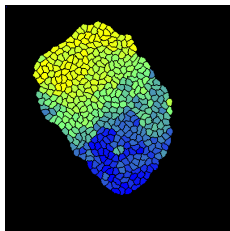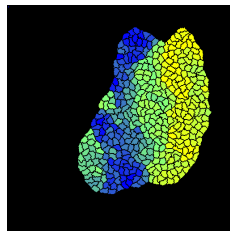

Supplement: S5 Fig — The graph shows how the adhesion proteins are distributed in the tissue, and the corresponding cell colour. The images show the tissue at the end of the simulation (2,000,000 MCS) for varying strengths of the maximum adhesion difference mm, without or with persistence (parameters μ: 2.0 and s: 30–40). J c, m = 15, maximum J i, j = 28. (PDF) [file pcbi.1004092.s005.pdf]

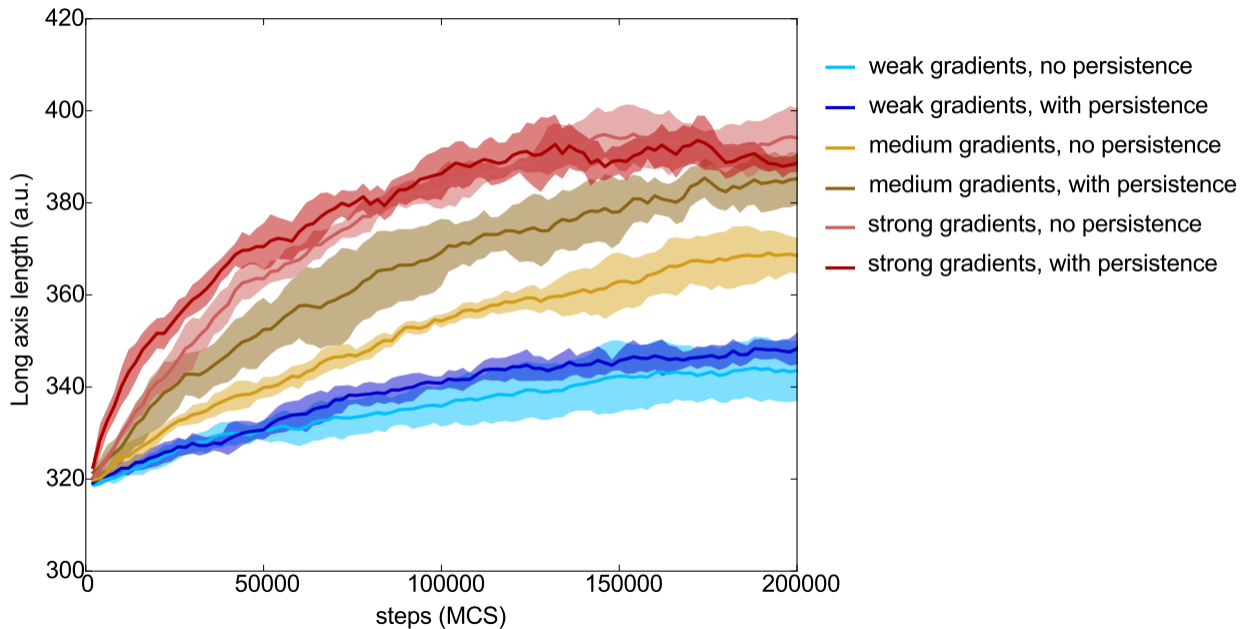

Supplement: S6 Fig — The graph plots the length of the long axis of the tissue over simulation steps for varying values of the maximum adhesion strength (mm: 12, 18, 24), and without or with persistence mechanism (parameters μ: 1.0 and s: 10).J c, m = 15, maximum J i, j = 28. (PDF) [file pcbi.1004092.s006.pdf]
