# Supplementary material for: Segment-Specific Adhesion as a Driver of Convergent Extension
Source: PLoS Comput Biol. 2015 Feb 23;11(2):e1004092. doi: 10.1371/journal.pcbi.1004092 (PMC4338282; doi:10.1371/journal.pcbi.1004092)
Supplement: S1 Table — (PDF) [file pcbi.1004092.s013.pdf]

$\gamma_{c,m} \rightarrow$ $\gamma_{r,g}$ $\downarrow$	2	6	10	14
2	10,18,16	14,18,16	18,18,16	22,18,16
6		12,18,12	16,18,12	20,18,12
10		10,18,8	14,18,8	18,18,8
14			12,18,4	16,18,4

Supplementary table 1. J values of the parameter space of figure 3 and supplementary figure 2.
The order of the values is $J_{c,m}$, $J_{r,g}$, $J_{r,r}$.
