# Supplementary material for: Segment-Specific Adhesion as a Driver of Convergent Extension
Source: PLoS Comput Biol. 2015 Feb 23;11(2):e1004092. doi: 10.1371/journal.pcbi.1004092 (PMC4338282; doi:10.1371/journal.pcbi.1004092)
Supplement: S1 Text — This document explains how the equilibrium contact length between two segments can be calculated from the area of the segments and the surface tensions. (PDF) [file pcbi.1004092.s014.pdf]

On surface tensions and segment interfaces

In the differential adhesion hypothesis, tissues are assumed to behave like immiscible fluids. When two drops of liquid have a positive interfacial tension, they reduce the contact area with each other; there is a force pulling inwards on the interface and the drops will round up. For a drop of liquid l on a surface s and in contact with the environment/air/medium e, Young's equation tells us that when the equilibrium state (minimum free energy) is reached, the contact angle θ of the drop with said surface is given by the surface tensions involved (see figure A below, and e.g. Wikipedia - contact angle):

$$cos\,\theta = \frac{\gamma_{s,e} - \gamma_{s,l}}{\gamma_{l,e}}$$

Without gravity, a 2D droplet will form a circle cut off by the surface, which means that from this angle and the total area of the droplet, we can calculate the amount of equilibrium contact between the droplet and the surface. For this, we use the fact that the droplet is a segment of the circle for $\theta < \frac{1}{2}\pi$, and that it is a circle with a segment missing for $\theta > \frac{1}{2}$. The area of a segment is given by $\frac{1}{2}(\alpha - sin\,\alpha)r^2$, where α is the angle of the sector enclosing the segment. See e.g. www.mathsisfun.com/geometry/circle-sector-segment.html.

Please note that in our case, the area and the surface tensions are in arbitrary (model), not physical units, and we mainly use this to deduce droplet shapes and ratios. Below, r is the radius of the circle, A the area, L the amount of contact:

$$r = \sqrt{\frac{2\,A}{2\,\theta - sin\,2\,\theta}}$$

$$L = 2\,r\,sin\,\theta$$

This can be extended to the situation where there are two drops of liquid adjacent to each other, as is the case in our model (figure b). In our calculations we assume that these two liquids have the same surface tension with the medium $\gamma_{c,m}$ and a (positive) tension with each other $\gamma_{r,g}$. The angle then is given by:

$$cos\,\theta = \frac{-\gamma_{r,g}}{2\,\gamma_{c,m}}$$

The calculation of the length of the interface then remains the same. Note that θ is $\frac{1}{2}\pi$ for $\gamma_{r,g} = 0$. If $\gamma_{r,g} < 0$, the two liquids will mix instead, forming one homogeneous spherical blob (Glazier Graner, Phys. Rev. E 1993).

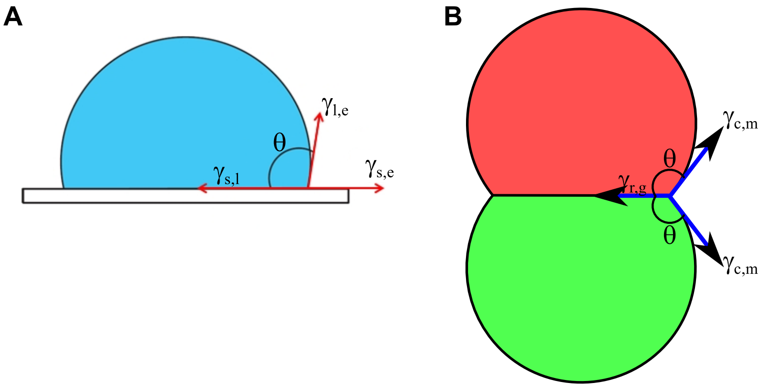

The preservation of aspect ratio for larger tissues

In the section concerning persistence, we assume that tissues of different sizes with the same number of segments and the same surface tensions, will end up with the same aspect ratio ($\frac{length}{width}$). This follows from the equations above. With the same surface tensions, the angles will be the same for the larger tissue, only the area of each segment is four times as large (we doubled both the length and the width of each segment). The radius of the circle then doubles, and so does the equilibrium contact length. In other words, if we start with a tissue that is twice the size in every dimension (so same aspect ratio), we end up with an elongated tissue with twice the equilibrium contact length, so still the same aspect ratio.
